# Optimising the community-based approach to healthcare improvement: Comparative case studies of the clinical community model in practice

**DOI:** 10.1016/j.socscimed.2016.11.026

**Published:** 2017-01

**Authors:** Emma-Louise Aveling, Graham Martin, Georgia Herbert, Natalie Armstrong

**Affiliations:** a Cambridge Centre for Health Services Research, University of Cambridge, UK; b Department of Health Policy and Management, Harvard T.H. Chan School of Public Health, USA; c Department of Health Sciences, University of Leicester, UK; d NIHR Biomedical Research Unit in Nutrition, Diet and Lifestyle at the University Hospitals Bristol NHS Foundation Trust and the University of Bristol, UK

**Keywords:** UK, USA, Improvement, Community-based, Clinical community, Case study

## Abstract

Community-based approaches to healthcare improvement are receiving increasing attention. Such approaches could offer an infrastructure for efficient knowledge-sharing and a potent means of influencing behaviours, but their potential is yet to be optimised. After briefly reviewing challenges to community-based approaches, we describe in detail the clinical community model. Through exploring clinical communities in practice, we seek to identify practical lessons for optimising this community-based approach to healthcare improvement. Through comparative case studies based on secondary analysis, we examine two contrasting examples of clinical communities in practice – the USA-based Michigan Keystone ICU programme, and the UK-based Improving Lung Cancer Outcomes Project. We focus on three main issues. First, both cases were successful in *mobilising diverse communities*: favourable starting conditions, core teams with personal credibility, reputable institutional backing and embeddedness in wider networks were important. Second, top-down input to organise regular meetings, minimise conflict and empower those at risk of marginalisation helped establish *a strong sense of community and reciprocal ties*, while intervention components and measures common to the whole community strengthened peer-norming effects. Third, to *drive implementation*, technical expertise and responsiveness from the core team were important, but so too were ‘hard tactics’ (e.g. strict limits on local customisation); these were more easily deployed where the intervention was standardised across the community and a strong evidence-base existed. Contrary to the idea of self-organising communities, our cases make clear that vertical and horizontal forces depend on each other synergistically for their effectiveness. We offer practical lessons for establishing an effective balance of horizontal and vertical influences, and for identifying the types of quality problems most amenable to community-based improvement.

## 1 Introduction

Securing improvements in healthcare quality is challenging (Powell et al., 2009). Even where interventions prove successful in one context, attempts to replicate positive impacts elsewhere are variable in their results (Dixon-Woods et al., 2013), and often disappointing (Lomas, 2005). Context is now recognised as a crucial mediator of efforts to improve healthcare quality, not just an inert backdrop, and furthermore one that can interact with interventions and implementation in unpredictable ways (Bamber, 2014). Accordingly, recognition has grown of the need to approach improvement interventions from a broader cultural and institutional perspective, accounting for the role of organisational structures and social processes (Aveling et al., 2013) and developing approaches to implementation that can adapt to contextual modifiers in more dynamic ways. In this context, the potential of community-based approaches is receiving increasing attention (Greenhalgh et al., 2010).

One potentially valuable feature of communities is an efficient, low-cost infrastructure for transmitting knowledge and innovation, including tacit knowledge or ‘know-how’ (Powell, 1990). A second is their power to shape behaviour through peer influence and normative pressures. Communities typically display strong shared identity and interdependence between members and may be especially powerful as ‘economies of regard’ (Offer, 1997), where peer sanctions and endorsements form a valuable currency. Evidence suggests threats to peer esteem and reputation in such communities may be more effective than formal hierarchical or legalistic efforts (Freidson, 1984). Thus, in contrast to legal or hierarchical-bureaucratic approaches, community-based approaches foreground the value of ‘horizontal’ links among peers, and the power of ‘bottom-up’ social processes driven by those peers rather than by leaders or managers at the apex of a hierarchy (Aveling et al., 2012a). Communities therefore offer an infrastructure for efficient knowledge-sharing and a potent means of influencing behaviours. Empirical studies suggest, however, that community-based approaches to healthcare improvement have yet to be optimised (Gabbay et al., 2003, Li et al., 2009, Nadeem et al., 2013).

We begin by reviewing some of the challenges encountered using community-based approaches to healthcare improvement, focusing on two prominent models: communities of practice and quality improvement collaboratives. We then describe a third model: the clinical community (Aveling et al., 2012a). This model builds on many of the principles of the first two, but also has distinctive features which, we argue, may address some of the challenges that have dogged community-based approaches thus far. Following this, we present a comparative analysis of two case-study clinical communities: the Michigan Keystone programme (USA) and the Improving Lung Cancer Outcomes Project (UK). In so doing, we identify practical lessons for optimising the clinical community approach.

### 1.1 Current approaches: communities of practice and quality improvement collaboratives

Two of the most well-developed community-based models for healthcare improvement are the ‘community of practice’ and the ‘quality improvement collaborative’. Communities of practice were first developed in the business sector and, as originally described (Lave and Wenger, 1991), are emergent and self-forming, centre on a shared concern or interest, and emphasise learning through practice—though further iterations since this original formulation have highlighted the role for managerial intervention in ‘nurturing’ communities of practice (e.g. Smith and McKeen, 2004). The quality improvement (QI) collaborative model developed within healthcare itself, with the Institute for Healthcare Improvement's ‘Breakthrough Series Collaborative’ model a well-known example. QI collaboratives form around specific, predetermined objectives and are typically time-limited. They characteristically use specific methods (such as PDSA cycles) and regular face-to-face (or sometimes virtual) events for learning and mutual encouragement (Hulscher et al., 2012, Nadeem et al., 2013). The evidence base for both models is somewhat mixed (Gabbay et al., 2003, Iedema et al., 2005, Li et al., 2009, Nadeem et al., 2013, Powell et al., 2009). Evidence reviews suggest that QI collaboratives can have some impact on quality of care processes but with marked variation in effectiveness (Nadeem et al., 2013), and that there is little evidence for the impact of communities of practice, partly because the model has been realised in very diverse ways (Li et al., 2009). Thus while some successes have been reported, these models are not without weaknesses.

First, critiques suggest that mobilising the diverse community of stakeholders needed to make improvements (typically including practitioners, managers and patients) (Aveling and Martin, 2013, Aveling et al., 2012a) can be problematic. Communities of practice assume the existence of a shared concern (a quality gap) around which healthcare practitioners will self-organize. Yet healthcare systems are frequently marked by historically embedded boundaries between professions, disciplines or organisational units, which create obstacles to knowledge sharing and a sense of shared interest (Ferlie et al., 2005). Further, quality gaps are often identified by external groups, and practitioners may disagree over their existence or importance. Collaboratives have also been found wanting in terms of mobilisation: though they may encourage and maintain enthusiasm among a motivated group of individuals, it is less clear that they can engage those less directly affected by change, whose cooperation is nonetheless required (Ayers et al., 2005, Benning et al., 2011, Carter et al., 2014).

A second challenge concerns promoting the shared sense of community and purpose needed to maintain cohesion and momentum (Aveling et al., 2012a). Both models tend to rely on their members’ goodwill, assuming that communities are naturally harmonious and egalitarian, when in fact they are typically fragmented and conflicted (Li et al., 2009). Discussion and decision-making can be undermined when professional or individual interests are allowed to dominate (Gabbay et al., 2003). Yet the communities of practice literature says little about how hierarchical or exclusionary dynamics can be avoided (Li et al., 2009).

Finally, current approaches have encountered difficulties generating and sustaining action. Whereas communities of practice are expected to instigate plans for change in the course of their own knowledge-exchange processes (Iedema et al., 2005), QI collaboratives are explicitly goal-focused from the outset, which arguably gives them an advantage in achieving improvement-related outcomes (Ayers et al., 2005). Even so, they still rely primarily on ‘volunteerism’ and, in a context replete with competing demands, volunteerism alone may prove insufficient to secure sustained action (Aveling et al., 2012a). Even when the energy and motivation of the membership is maintained, communities often struggle to turn plans into action because they lack the necessary resources, expertise, skills or leadership and direction (Li et al., 2009, Øvretveit et al., 2002).

### 1.2 Clinical communities

The model of the clinical community (Aveling et al., 2012a) is relatively new and was developed through a detailed literature review. While sharing many of the basic principles of communities of practice and QI collaboratives, it also incorporates some distinctive features which seek to tackle the shortcomings just identified (i.e. around mobilisation, sense of community, and sustained action).

A clinical community is formed of interdependent individuals, united by a shared commitment to specific goals, who work collaboratively to achieve these. It has reasonably well-defined boundaries (to mitigate loss of focus or identity), which are porous enough to transcend organisational, disciplinary and professional boundaries to ensure inclusion of the necessary stakeholders. Clinical communities are distinguished by a ‘vertical’ integrating core to complement reciprocal ‘horizontal’ relationships between peers. The core leads organisation of the community and its resources, and ensures sustained direction and coordination. The core also plays an important role in mobilising an inclusive community of stakeholders; given the challenges described above, this may mean persuading and engaging skeptics. Recognising the limits of relying on ‘volunteerism’ in healthcare contexts crowded with competing priorities, sometimes the vertically integrating core may deploy harder tactics, by which we mean those that are more directive, enforcing and even coercive (Vangen and Huxham, 2003), alongside persuasion to ensure sustained focus and action. To this extent, clinical communities seek to explicitly address one of the tensions identified for communities of practice: that while managerial intervention can result in the ‘dissipation’ of a community (Smith and McKeen, 2004), communities also require “specific managerial efforts to develop them and integrate them” with wider organisational efforts (Wenger and Snyder, 2000). Clinical communities incorporate such concerted, purposeful intervention right from the start.

Unlike communities of practice, clinical communities are a forum for change as well as knowledge-sharing. And unlike collaboratives, the strategies and activities used to deliver change are not defined by the model itself. The clinical community model is therefore perhaps best conceptualised as an improvement architecture. Specific elements such as choice of technical intervention to be delivered by the clinical community may vary, but the structure – a vertically-integrating core (as described above) and horizontal links between members – does not. The central dynamic of the clinical community is therefore not purely the collective power of horizontal peer-to-peer relationships (as with other models), but a balance of both these vertical and horizontal influences. This is a difficult balance to strike, however. On the one hand, the vertically-integrating core must provide adequate direction and resources and have sufficient hard tactics at its disposal to keep its members on track. Yet too much ‘top-down’ input risks establishing an essentially hierarchical approach, undermining the power and unique advantages emanating from the *horizontal* links developed. Equally important, therefore, is establishing strong, reciprocal ties between members, which heighten ‘social observability’ and increase opportunities for community norms to be established (Holtman, 2008). In turn, this maximizes the potential for informal, social control mechanisms (e.g. peer norming effects) to sustain focus and drive action. Similarly, members are more likely to continue to devote time and energy if they share a strong sense of community and a cohesive, shared identity (Gillespie et al., 2008). Although properties of the horizontal links of a community, these characteristics are unlikely to be self-forming within the healthcare context – traditionally marked by divisions, hierarchies and competing priorities – and as such require directive input.

While the theory underpinning the clinical community model has been set out in some detail (Aveling et al., 2012a), how to deploy it effectively is less well understood (though see Gould et al., 2015 for some examples). We seek to advance this through comparative analysis of two case studies, focusing particularly on lessons for developing effective vertical and horizontal structures and striking the right balance between them.

## 2 Methods

Comparative case studies (Yin, 2003) using secondary analysis (Campbell and Cornish, 2012) form the basis of our methodology. We have been involved – in different ways – in studying both cases presented. To conduct a comparative secondary analysis of these projects, we use our publications, other reports and published accounts, as well as reflections from our involvement in studying them (cf. Campbell and Cornish, 2012).

We take this approach because comparative study enables analysis to move beyond description of individual cases to the development of more generalizable theoretical insights (Druckman, 2005). Through such theorisation we seek to enhance understanding of how to optimise this model in practice. In one sense our *selection* of cases was pragmatic, as there are few clinical-communities-in-practice to our knowledge. But our selection is also valuable because the cases are dichotomous: that is, while both deployed the clinical community architecture as defined above, they differed in important ways, notably the clinical area targeted, specific QI activities and strategies used, and the degree of standardisation. The cases also differed in their success in achieving significant improvements in outcomes for patients. Most notably, the cases differed in the way they responded to key challenges facing community-based approaches—mobilisation, sense of community and sustained action—as our analysis will show.

Dichotomous case selection allows new insights to be generated through exploration of the reasons for and consequences of inter-case differences (Schensul et al., 1999). Thus our comparison of cases was guided by the challenges for community-based approaches reviewed above, around mobilising inclusive communities, promoting and sustaining a strong sense of community, and ensuring plans are turned into action. We focused on identifying practical lessons about whether and how the cases were able to meet these challenges, particularly through exploration of divergence and contrast between them.

### 2.1 Michigan Keystone ICU programme

Our account of the first case—the Michigan Keystone ICU programme – draws on published literature, cited where appropriate. We draw particularly on an ex-post theorisation accounting for the programme's outcomes (Dixon-Woods et al., 2011) developed by three social scientists (including ELA) in collaboration with programme leaders.

The Michigan Keystone ICU programme (henceforth ‘Michigan’), conceptualised as a ‘clinical community’ (Pronovost et al., 2013), engaged intensive care units in Michigan, USA, to implement a multifaceted intervention to reduce central venous catheter-related bloodstream infections (CVC-BSIs). Michigan's vertically-integrating core comprised two critical care clinicians, and the programme was hosted by the Michigan Hospitals Association.

Following a state-wide invitation, 103 ICUs (covering 85% of ICU beds) signed up and completed the programme (Pronovost et al., 2006). Participating teams each included a senior manager (e.g. hospital executive), an infection-control practitioner and two ‘frontline’ team leaders – a physician and a nurse. Each participating team was required to implement a series of standardised cultural and technical interventions directed at reducing CVC-BSIs, although teams could choose the order of interventions and make some local adaptations (e.g. formatting of tools).

Team leaders received training on safety science and the intervention's components through regular conference calls, coaching and biannual state-wide meetings. Each team also received a written package summarising the evidence for the intervention components, suggestions to guide implementation, and instructions for data collection. Locally, team leaders were expected to cascade information to colleagues and, with support from local managers, the core team and the wider community, implement the intervention components. Data were collected monthly and submitted to the core team; the number and rate of CVC-BSIs were then fed back to participating teams monthly.

The programme received international acclaim for its success in achieving a sustained reduction in CVC-BSIs: the median rate of infection per 1000 catheter-days decreased from 2.7 to 0, sustained over 15 months’ follow-up (Pronovost et al., 2006).

### 2.2 Improving Lung Cancer Outcomes Project

All authors were involved in studying the second case—the Improving Lung Cancer Outcomes Project—through two commissioned evaluations of the programme. This analysis draws on evaluation reports and other published articles about the project (cited as appropriate).

The Improving Lung Cancer Outcomes Project (henceforth ‘ILCOP’), was funded by the Health Foundation. It entailed a multifaceted intervention to improve lung cancer outcomes in 30 National Health Service hospitals in the UK. ILCOP's vertically-integrating core comprised a clinical lead (chest physician), a full-time project manager and a quality improvement facilitator; a wider steering group included representatives of lung cancer charities, cancer nurse specialists, the Royal College of Physicians (RCP), and the National Lung Cancer Audit (NLCA).

All 152 lung cancer multi-disciplinary teams (MDTs) in England were invited to participate; 91 agreed and 78 were ultimately included (Russell et al., 2014). Participating MDTs identified a minimum required membership (physician, nurse specialist and MDT coordinator). MDTs were formed into pairs, with 15 pairs randomised to the intervention group (i.e. ILCOP participation), and the rest controls.

Each participating MDT engaged in a programme of activities organised by the core team. First, reciprocal peer-review visits between pairs facilitated by the quality improvement facilitator (Aveling et al., 2012b). During visits, strengths and weaknesses of the host MDT's provision were identified through observation of its meeting and discussion of its NLCA data, patient experience data and other information. The project also involved: development of locally-tailored Quality Improvement Plans (QIPs), targeting a specific area of service provision, using a QIP template and feedback from the core team; three national workshops; and online conferences hosted by the core team to discuss good practice and common challenges. The quality improvement facilitator provided on-going technical support to individual teams, connecting them with individuals in the steering group or other sites with relevant expertise where appropriate.

Eighteen teams collected local data to measure the impact of QIP implementation, some of which showed improvements in the targeted areas. ILCOP's core team also used NLCA data to measure the project's overall impact. NLCA indicators improved similarly in the intervention and control groups, with the exception of the proportion of patients receiving active anti-cancer treatment, which increased by 5.2% in the intervention group versus 1.2% in the control (Russell et al., 2014).

### 2.3 Findings

We present our findings in terms of whether and how the case studies met three key challenges for community-based approaches: mobilising a diverse community; establishing a strong sense of community and reciprocal ties between members; and ensuring plans are implemented and momentum sustained. In drawing out practical lessons, we focus particularly on the central, distinguishing feature of clinical communities: the combination of horizontal and vertical structures.

### 2.4 Mobilising the community

Both cases were successful in mobilising diverse communities, securing participation of multidisciplinary teams in a high proportion of sites. Both areas of healthcare represented relatively favourable starting conditions due to the existence of established networks: English regional cancer networks, and the Michigan Hospitals Association (MHA) network. Core teams used these to engage potential participants. Both programmes were able to harness isomorphic pressures, whereby over time some members felt compelled to put themselves forward for participation as recruitment gained momentum. Common regulatory and reputational pressures may have encouraged the Michigan ICUs to mimic one another (Dixon-Woods et al., 2011), while the UK lung cancer community was relatively small and integrated. The ability to utilise these networks effectively reflected the composition of the core team, notably their affiliations with multiple organisations, professions and stakeholder groups.

Equally important was the credibility—amongst practitioners in particular—of the programmes’ leadership, and their institutional backing. Both leadership teams included clinicians, some of whom were recognised for previous, successful improvement efforts. As a result, both programmes enjoyed legitimacy as experts in improvement. Both programmes also had affiliations with prestigious, respected and influential organisations – Johns Hopkins Medical School and the MHA for Michigan, and the RCP (a professional society) for ILCOP—conferring further credibility.

At site level, Michigan sought to secure an inclusive community by requiring a letter of commitment from hospitals identifying site-level team members, including practitioners and management. ILCOP required sites to identify a ‘minimum’ team (physician, cancer nurse specialist and MDT coordinator), building on previous work by the RCP and others to establish multi-disciplinary working in this area.

Communities also need a uniting vision to mobilise *around* (Aveling et al., 2012a). To make the case for improvement, ILCOP relied largely on NLCA data showing UK lung cancer outcomes were worse than elsewhere in Europe, and had improved less than other cancers in the UK. The national audit set benchmarks (largely already accepted by practitioners as legitimate standards), thereby providing a focus for ILCOP participants to unite around. In order to establish a consensus that a problem existed in ICUs, and that CVC-BSIs were not inevitable in critical care, Michigan combined patient stories with data demonstrating variability in infection rates across ICUs. These tactics helped to promote a common cause around which stakeholders could mobilise and create a uniting vision (Dixon-Woods et al., 2011).

### 2.5 Promoting and sustaining a strong sense of community

In both programmes, meetings and virtual events were key strategies to develop horizontal links between teams who would otherwise have little contact. Such events also provided a rare opportunity for protected time with colleagues to plan and reflect on improvement work and build social bonds *within* teams. Regular activities also helped to maintain local teams' focus and enthusiasm, given that the interventions were often an ‘added extra’. Making these activities into an effective means of community-building took work, however—by organising community activities, core teams minimised the logistical burden, making it easier for busy practitioners to take time out of hectic schedules. This logistical and administrative work was carried out by paid, core team members with dedicated time.

Some vertical, top-down input also proved necessary to help manage divisions and power asymmetries within participating sites. In ILCOP, peer review visits were carefully structured and planned; structuring discussion to include direct peer-to-peer (e.g. nurse-to-nurse) discussion before whole-group discussion helped strengthen the voice of groups at risk of marginalisation, and raised some individuals’ confidence in contributing to subsequent group discussions. Michigan sought to ensure inclusion and minimise conflict through a combination of allocating responsibilities, facilitating collective agreement of rules and responsibilities, and providing third-party facilitation where necessary. For example, nurses were charged with monitoring checklist implementation and halting procedures if appropriate steps were not followed. To enable this contravention of a traditionally hierarchical relationship, the programme empowered nurses by writing this duty into *collectively* agreed rules, and giving nurses access to third-party leaders (hospital executives or the core team) with greater leverage over doctors. Although the core team could not intervene to resolve all local-level conflicts, these strategies did help to tackle unhelpful hierarchical dynamics not only during planning, as in ILCOP, but also during implementation.

While the functions of collective activities were similar across the two programmes, ILCOP and Michigan achieved different levels of success in strengthening the horizontal ties that sustained action. While in Michigan participating teams began to lead group discussions and support each other directly, in ILCOP most teams relied on relationships with the core team as their principal support. One point of contrast that contributed to this difference was that the Michigan team had clear plans (and allocated resources) from the outset for comprehensive community-building activities. Regular teleconferences supported frequent interactions and reciprocal communication amongst large numbers of participants throughout the intervention; this was increasingly participant-led, reducing dependency on the core team. In addition, twice-yearly two-day *residential* gatherings, including a ‘cocktail hour’ and unstructured social time, were important in reinforcing relationships and sharing know-how amongst peers.

In contrast, building a sense of community amongst ILCOP participants was not initially an explicit part of the core team's plans; online workshops and two half-day workshops (at the mid-point and end of the programme) were added later. As in Michigan, participants valued the combination of formal sessions and informal socialising. But face-to-face gatherings were infrequent (only two during the course of the programme), and, in contrast to Michigan, ‘virtual’ events had poor attendance (1–5 participants). In part this may have been because these were time-consuming activities to which participating teams had not originally committed, and, unlike in Michigan, ILCOP's local leads did not have dedicated project time for such activities.

Another factor contributing to the divergent sense of community was how each programme sought to ensure teams retained professional ownership of improvement work. Michigan already had an established ‘solution’ to propose: the core team's task was to ensure it was accepted by the community. Their ability to establish consensus on the proposed intervention was bolstered by a strong evidence base. These starting conditions were reinforced by the initial teleconference calls which provided opportunities for debate and challenge so that teams could raise doubts, clarify expectations, establish shared understanding of the intervention and what constituted ‘success’. Crucially, this discussion and consensus-building happened *before* teams started technical interventions.

In contrast, ILCOP used dialogue and exchange among peers during reciprocal peer review visits to enable each participating team to identify and decide for itself which area(s) to target and how to go about it. In many ways these peer review visits proved effective forums for identifying weak areas of performance (Aveling et al., 2012b). However, this approach also meant that most teams had different ‘targets’ for improvement and used different strategies. Thus while both programmes were successful in ensuring *local* ownership, in ILCOP the extent to which this sense of ownership took a form common to the whole community was limited: in contrast to Michigan's set of centrally agreed targets and strategies, each ILCOP team was ‘doing its own thing’, curtailing opportunities for comparison and peer norming effects.

### 2.6 Moving from intention to implementation

For many community members, quality improvement was a new exercise of which they had no prior experience. Both core teams played a central role in supporting members in this regard. Both included members with expertise in quality improvement, the relevant evidence-base, and data systems. In addition to helping teams rectify problems with validation of NLCA data (important in identifying local intervention targets), ILCOP provided templates for QIPs and offered expertise in the areas of practice teams were targeting. In Michigan, teams were given ‘toolkits’ covering all aspects of the programme, including guidelines, team roles and responsibilities, best-practice protocols, evidence summaries and supporting materials for implementation (e.g. data collection forms). In addition, regular teleconferences meant the expertise amongst members was shared with peers.

Another valuable feature of the core teams was that they were streamlined, enabling quick and efficient decision-making. Both had a small, ‘inner core’ of 2–3 members who met frequently; both core teams retained decision-making powers allowing them to adapt programme design as needed. Equally important was remaining responsive to developments ‘on the ground’—Michigan through regular teleconferences, ILCOP through the cancer networks in which it was embedded, and the QI facilitator and project manager. Examples of this responsiveness included a targeted programme of follow-up support for teams during the implementation phase (ILCOP), and adapted project materials following discussions with participating teams (Michigan).

Core teams could not afford to be too responsive, however. Ensuring community members remained committed to improving outcomes for patients sometimes required the employment of hard tactics. While both programmes found the need to use a range of strategies, including softer options such as persuasion and ongoing discussion, Michigan had a greater range of hard tactics at its disposal and was more directive than ILCOP throughout.

Michigan built a number of hard tactics into programme design from the outset, primarily through its more standardised intervention and making participation conditional on minimum commitments from the team *and* hospital CEOs. Before a unit could enrol in the programme, the team had to agree, for example, to submit data. Failure to do so would result in the team being asked to leave the programme. Hospital CEOs had to submit letters of commitment agreeing to, *inter alia*, an ICU physician and an ICU nurse devoting a specified proportion of their time to the intervention, and creation of a dedicated ‘central line cart’ resourced with the necessary equipment for safe CVC insertion. Through such hard tactics, the Michigan core team tried to minimise potential *local* obstacles to implementation for members.

ILCOP was less directive in what it requested: although sign-off from CEOs was mandatory, no minimum requirements were specified. Over time, tactics such as persuasion, information provision and reminders proved insufficient. For example, getting all teams to submit a QIP required extensive chasing, and getting them to return data about their interventions was even more difficult (only 18/30 ultimately did so). The lack of available hard tactics also increased the burden for local team leads: for example, some teams struggled to get support from local managers with control over resources, or cooperation from other departments needed to implement a QIP.

Michigan could also be more directive than ILCOP because its programme elements were more standardised. In part standardisation was possible because CVC-BSIs represented a well-bounded issue for which interventions with an established evidence base existed. This enabled the core team to build-in potential hard tactics more easily: the non-negotiable (evidence-based) elements of a standardised intervention package; the agreement that empowered nurses to contact executives if physicians were not complying; signed checklists of mandatory practices, which created an auditable trace of adherence (Dixon-Woods et al., 2011). Simultaneously, the core team encouraged customisation of certain elements to fit with local culture and resource availability. For example, teams could design their own checklist format. Michigan, then, sought to maintain a sense of local ownership by allowing customisation of the ‘how’ of intervention, but not the evidence-based content of the interventions (the ‘what’).

In allowing each team to choose their focus for improvement and devise their own QIPs, ILCOP incorporated many different, localised interventions. While this secured local ownership and sensitivity, it also meant that the core team could not set many minimum requirements at the time of sign-up, since what teams' chosen interventions would entail was unknown. The complex nature of lung cancer care also meant that the range of potential stakeholders was far greater, with many team members (e.g. pathologists and radiologists) also supporting other clinical areas. It was therefore more difficult for the core *and local* teams to secure the support needed when QIPs tackled problems that involved these more peripheral MDT members. A further difficulty was that, partly due to the heterogeneity and complexity of lung cancer pathways, ILCOP did not have an evidence-based formula that would reliably fit different hospitals. Locally-designed QIPs varied in quality, and some were less ambitious than was perhaps necessary to measurably impact clinical outcomes. The core team did provide feedback, but to avoid undermining their commitment to local ownership this sometimes required delicate negotiation, and, in the absence of pre-agreed commitments to local measurement to drive and refine improvement, there was some drift in local definitions of ‘success’ (Aveling et al., 2012b). This indicates that while professional ownership is important, local customisation needs to be contained within defined limits to avoid being at the expense of meaningful changes in practice.

It is important to recognise, however, that the potential value of hard tactics did not only derive from rules set ‘vertically’ by the core team. Crucial to their efficacy in most cases was a mutually-reinforcing interplay between vertical and horizontal pressures, and the way compliance with (or progress towards) standardised elements of the programme could become ‘normed’ through the horizontal links and ‘bottom-up’ influence of the community. This is best illustrated through our cases' use of data to drive implementation and action.

Data collection and feedback has specific advantages within community-based approaches (Aveling et al., 2012a): sharing and comparing data aids the establishment of shared norms and harnesses peer influence. Collecting data is typically very challenging, though. Both core teams provided participants with valuable support regarding the technical aspects of data collection, and could rely (at least in part) on existing measures for which a nationally-supported infrastructure already existed (Beckett et al., 2012, Pronovost et al., 2006). More interesting is what the contrasts between our cases reveal about how to effectively harness the potential of data to drive, refine and sustain improvement work.

In Michigan, teams spent three months collecting baseline data, refining data-collection systems, and addressing training needs to ensure data completion and quality. This process helped to establish consensus on the data's validity, countering concerns about credibility. Standardised data, comparable across all sites, was fed back regularly to motivate and sustain efforts (number of infections monthly, rate of infections quarterly). Teams were also provided with blinded data from the rest of the programme to assess their progress relative to others. This helped keep members ‘on task’ and connected to the community, and could stimulate poorer performers to try to improve. Thus although mandatory data submission represented a vertical ‘push’ from the core team, its effectiveness stemmed from horizontal, normative pressures within the clinical community.

At programme level, ILCOP made good use of routinely collected NLCA data, which most teams already collected and which was seen as credible. But while NLCA would ultimately provide robust outcome data for the programme, it was less useful during the *process* of improvement. NLCA data showing programme outcomes was not collated and released until over a year after the programme finished, and could not therefore be used to motivate efforts or harness peer pressure. ILCOP did request that teams identify local measures for each QIP, but only 18 of 30 complied. Some teams were unwilling or unable to collect data over and above that required for mandated national audits, not least since many did not realise they had signed up to local measurement. Where local measurements were returned, the peer norming effects were limited since different teams were doing and measuring different things.

Another approach to driving implementation used in both cases was to align improvement efforts with drivers within the wider policy context. Michigan did this, for example, by garnering the support of one of the region's largest insurers. The insurer incentivised hospitals financially to participate and improve their infection rates. Our comparison illustrates that such alignment tactics can be double-edged, however. In the UK, some standards assessed through NLCA were aligned with national targets with financial implications for the hospital; others were not. Where minimum standards were already met but teams wanted improvement, there was no financial incentive to motivate managerial support for ILCOP-related projects (Martin et al., 2015).

Not all obstacles to implementation could be solved by the core teams, especially contextual issues relating to the particularities of individual sites. In ILCOP, mergers or service re-organisation in some hospitals caused tensions and divisions that deterred staff from engaging. Similarly, some Michigan teams found it difficult to engage physicians. The social and political skills of local leads therefore remained critical, regardless of the positive influence of the clinical community. More general contextual issues, such as availability or distribution of resources within the system, also posed problems. In Michigan, larger hospitals found it easier to divert resources to the programme; in ILCOP, financial difficulties in some hospitals meant teams struggled where their chosen improvements required additional resources.

## 3 Discussion

Many of the challenges our case studies encountered are not unique to clinical communities; several are common features of other community-based approaches (e.g. establishing an inclusive, cohesive community) and improvement initiatives more broadly (e.g. data collection and sustaining focus). Our case comparison helps to identify some practical ways in which these challenges can be addressed by clinical communities. It also generates lessons about the types of quality problem and intervention context that may be tractable to a community-based approach.

Contrary to the idea of self-organising communities, a vertically integrating core team (capable of being directive and deploying hard tactics where needed) proved vital to the operation of horizontal forces within both communities. Our cases make clear that vertical and horizontal forces do not simply counter-balance each other; rather, they depend on each other for their effectiveness in a synergistic way. Vertical input and hard tactics were at their strongest when the sense of community and horizontal links were also strong; equally, vertical forces played an important role in cultivating the dense horizontal ties that give a community-based approach its force. For example, in Michigan many core-team interventions helped to strengthen horizontal ties: events that allowed social bonds to develop; stories that helped mobilise the community, offering a shared sense of purpose; requirements for inclusive teams; and standardised but collectively agreed rules, intervention components and data. In turn, the strong sense of community amplified the disciplining effects of top-down directives such as mandatory submission and sharing of data.

Our comparison suggests that effective, vertical structures require: leaders with credibility and legitimacy; connections with existing networks and reputable healthcare institutions; a complement of paid staff able to dedicate time to the heavy demands of organising and running a programme; and structures and processes that enable the core team to be responsive and remain in touch with participants. At the same time, our analysis shows that incorporating into programme design sufficient hard tactics to enable core teams to be directive or coercive when needed is important. These might include: minimum requirements for enrolment with consequences for non-compliance; agreements that leverage the influence of more powerful individuals within institutions; inclusion of a non-negotiable core of intervention elements; cautious alignment with wider system incentives.

Directive input from core teams could not overcome all obstacles, however, including those posed by contexts of significant resource constraint or service re-organisation. The need for skilled, local leads to engage colleagues, identify local policy levers and leverage resources remained crucial. Yet in reality, improvement programmes are unlikely to have the option of choosing between more or less effective leaders at the local level. Building in hard tactics helps to minimize the burden on (variably effective) local leads.

Our analysis also suggests that standardisation of the intervention and data collection processes helps capitalise on the potential power of horizontal forces for driving change, while furnishing some hard tactics that can be deployed to keep teams on track. Michigan had an advantage here: its more standardised intervention and data collection processes facilitated directive input where needed, while also allowing comparison and a shared sense of ownership. Controls on the extent of this must be in place to maintain quality of the intervention and comparability of performance. As the ILCOP experience showed, giving too much control to local teams over what to tackle and how can undermine the impetus for sustained action deriving from a strong sense of community ownership. The Michigan case suggests that one promising approach is to allow customisation on the ‘how’ of implementation and the format of tools, but not on the standards of practice or the content of tools (the ‘what’).

The need for some degree of standardisation of measures, intervention targets and data collection processes at the community level also suggests that some types of quality problem are more amenable to community-based improvement than others. ILCOP may have struggled to secure consistent improvements not only due to programme design and realisation, but also due to intrinsic features of the clinical problem being tackled. While Michigan tackled a relatively bounded phenomenon (insertion and management of central lines), managed within a single unit (the ICU) that was fairly uniform across sites, lung cancer services are complex pathways spanning multiple units organised in highly variable ways. In addition, while there was a clear evidence base for Michigan's technical intervention, improving lung cancer outcomes was in a more exploratory phase, where there was often no evidence for the steps required to improve particular outcomes. Consequently, regardless of programme design, it would have been hard to achieve consensus on a standardised intervention for use by the whole lung cancer community. In addition, the Michigan intervention required changes in norms and behaviours around a specific practice, whereas improvements in lung cancer care sometimes entailed structural changes or complex coordination between multiple departments only partially focused on lung cancer care.

Thus it may be that clinical communities are best-suited to quality gaps for which a clear evidence-based solution exists, so local customisation can focus on how to implement standardised steps, not what to implement. For problems without a clear evidence-based solution, local innovations have an important place (and indeed might be evaluated to produce a robust evidence base) but it is not clear that a clinical community is the best way of delivering or co-ordinating such locally-driven work. Similarly, clinical communities are best-suited to instigating improvements that depend primarily on changes in behaviour or culture within bounded areas (susceptible to norming effects), rather than large structural or resource-dependent changes.

And what of cases, such as ILCOP, where the quality gap identified is broader and more complex (lung cancer outcomes rather than CVC-BSI prevention), and against which the performance of local teams may be more nuanced (e.g. both good and bad in parts)—and so the necessary intervention is not self-evident and cannot be pre-ordained? One compromise might be allowing local teams to select both ‘what’ and ‘how’ but only from a defined menu of options, which are amenable to a common set of real-time measurements, to allow comparison across the community.

While we believe our comparative analysis of two clinical communities is valuable, we acknowledge certain limitations. First, although we have studied and published on both case studies, our previous work with ILCOP was through extended, real-time evaluation, while our work with Michigan was solely post-hoc. One consequence of this is that, in contrast to ILCOP secondary data, we had limited information on Michigan from participants' (as opposed to programme leads') perspectives, meaning we could explore in less depth the variation between sites, and community members' own perceptions of the strength of the shared sense of community. Second, the post-hoc Michigan analysis (Dixon-Woods et al., 2011) was initiated *because of,* and to explain, the success of the program, and so may have focused less on challenges or weak points (although it does acknowledge problems faced and adaptations made); in contrast the ILCOP process evaluation was conducted largely before outcome data were available. Third, while many of the differences between programmes are conducive to an interesting and fruitful comparison, it is perhaps not ideal that only one included a control group. These differences place limits on any assertions that the changes in outcomes recorded were directly attributable to each community's activities. Even so, the strength of secondary analysis in bringing together diverse and dichotomous cases offers a valuable opportunity to explore differences and their implications, in this case the practical implications for clinical communities-in-practice.

## 4 Conclusion

Central to the efficacy of clinical communities as an architecture for healthcare improvement is the dynamic, symbiotic tension between vertical and horizontal forces that drives commitment and action for change. A credible, well-embedded core team and the incorporation of directive, hard tactics into programme design are needed not only to drive change from the top, but also because they play a central role in cultivating the horizontal ties that give a community its power. Effective use of the clinical community approach does not only depend on optimising the model itself, however; the context and improvement objective for its application must also be carefully considered. Our study suggests its potential advantages are more likely to be realised where evidence-based interventions with some degree of standardisation across the community are feasible, and where change primarily depends on those aspects of healthcare delivery susceptible to peer-influence and norming effects.

## Funding and acknowledgements

Emma-Louise Aveling's contribution was supported by funding from a Wellcome Trust Senior Investigator award [WT097899]. Graham Martin's contribution was supported by the National Institute for Health Research (NIHR) Collaboration for Leadership in Applied Health Research and Care East Midlands (CLAHRC EM). The views expressed in this publication are those of the author(s) and not necessarily those of the NHS, the NIHR or the Department of Health. Previous work with the Improving Lung Cancer Outcomes project was funded by the Health Foundation, registered charity 286967, as part of its evaluation of the Closing the Gap through Clinical Communities programme.

The authors wish to thank the following: Mary Dixon-Woods for helpful comments on earlier drafts of this paper; Janet Willars for help with data collection; Liz Shaw for help with coding; Lisa Hallam for administrative support. We also wish to thank the leads of both the Michigan Keystone ICU programme and the Improving Lung Cancer Outcomes Project for their willingness to work with us.

## References

[bib1] Aveling E.L., Martin G.P., Armstrong N., Banerjee J., Dixon-Woods M. (2012). Quality improvement through clinical communities: eight lessons for practice. J. Health Organ. Manag..

[bib2] Aveling E.-L., Martin G., Jimenez Garcia S., Martin L., Herbert G., Armstrong N., Dixon-Woods M., Woolhouse I. (2012). Reciprocal peer review for quality improvement: an ethnographic case study of the Improving Lung Cancer Outcomes Project. BMJ Qual. Saf..

[bib3] Aveling E.L., McCulloch P., Dixon-Woods M. (2013). A qualitative study comparing experiences of the surgical safety checklist in hospitals in high- and low-income countries. BMJ Open.

[bib4] Aveling E.L., Martin G. (2013). Realising the transformative potential of healthcare partnerships: insights from divergent literatures and contrasting cases in high- and low-income country contexts. Soc. Sci. Med..

[bib6] Ayers L.R., Beyea S.C., Godfrey M., Harper D.C., Nelson E.C., Batalden P.B. (2005). Quality improvement learning collaboratives. Qual. Manag. Healthc..

[bib7] Bamber J. (2014). Foreword. Health Foundation, Perspectives on Context.

[bib8] Beckett P., Woolhouse I., Stanley R., Peake M. (2012). Exploring variations in lung cancer care across the UK–the ‘story so far’ for the national lung cancer audit. Clin. Med..

[bib9] Benning A., Ghaleb M., Suokas A., Dixon-Woods M., Dawson J., Barber N., Lilford R. (2011). Large scale organisational intervention to improve patient safety in four UK hospitals: mixed method evaluation. BMJ.

[bib10] Campbell C., Cornish F. (2012). How can community health programmes build enabling contexts for transformative communication? Experiences from India and South Africa. AIDS Behav..

[bib11] Carter P., Ozieranski P., McNicol S., Power M., Dixon-Woods M. (2014). How collaborative are quality improvement collaboratives: a qualitative study in stroke care. Implement. Sci..

[bib12] Dixon-Woods M., Bosk C.L., Aveling E.L., Goeschel C.A., Pronovost P.J. (2011). Explaining Michigan: developing an ex post theory of a quality improvement program. Milbank Q..

[bib13] Dixon-Woods M., Leslie M., Tarrant C., Bion J. (2013). Explaining Matching Michigan: an ethnographic study of a patient safety program. Implement. Sci..

[bib14] Druckman D., Druckman D. (2005). Comparative case study approaches. Doing Research.

[bib15] Ferlie E., Fitzgerald L., Wood M., Hawkins C. (2005). The nonspread of innovations: the mediating role of professionals. Acad. Manag. J..

[bib17] Freidson E. (1984). The changing nature of professional control. Annu. Rev. Sociol..

[bib18] Gabbay J., le May A., Jefferson H., Webb D., Lovelock R., Powell J., Lathlean J. (2003). A case study of knowledge management in multiagency consumer-informed communities of practice: implications for evidence-based policy development in health and social services. Health.

[bib40] Gillespie A., Cornish F., Aveling E.L., Zittoun T. (2008). Conflicting community commitments: a dialogical analysis of a British woman’s World War II diaries. J. Community Psychol..

[bib19] Gould L., Wachter P., Aboumatar H. (2015). Clinical communities at Johns Hopkins medicine: an emerging approach to quality improvement. JCJQPS.

[bib20] Greenhalgh T., Stramer K., Bratan T., Byrne E., Russell J., Potts H.W. (2010). Adoption and non-adoption of a shared electronic summary record in England: a mixed-method case study. BMJ.

[bib21] Holtman M.C. (2008). A theoretical sketch of medical professionalism as a normative complex. Adv. Health Sci. Educ..

[bib22] Hulscher M.E., Schouten L.M., Grol R.P., Buchan H. (2012). Determinants of success of quality improvement collaboratives: what does the literature show?. BMJ Qual. Saf..

[bib23] Iedema R., Meyerkort S., White L. (2005). Emergent modes of work and communities of practice. Health Serv. Manag. Res..

[bib24] Lave J., Wenger E. (1991). Situated Learning: Legitimate Peripheral Participation.

[bib25] Li L.C., Grimshaw J.M., Nielsen C., Judd M., Coyte P.C., Graham I.D. (2009). Use of communities of practice in business and health care sectors: a systematic review. Implement. Sci..

[bib26] Lomas J. (2005). Using research to inform healthcare managers' and policy makers' questions: from summative to interpretive synthesis. Healthc. Policy.

[bib41] Martin G.P., Armstrong N., Aveling E.-L., Herbert G., Dixon-Woods M. (2015). Professionalism redundant, reshaped, or reinvigorated? Realizing the ‘Third Logic’ in contemporary healthcare. J. Health Soc. Behav..

[bib27] Nadeem E., Olin S.S., Hill L.A., Hoagwood K.E., Horwitz S. (2013). Understanding the components of quality improvement collaboratives: a systematic literature review. Milbank Q..

[bib28] Offer A. (1997). Between the gift and the market: the economy of regard. Econ. Hist. Rev..

[bib29] Øvretveit J., Bate P., Cleary P., Cretin S., Gustafson D., McInnes K., Robert G. (2002). Quality collaboratives: lessons from research. Qual. Saf. Health Care.

[bib30] Powell W.W. (1990). Neither market nor hierarchy: network forms of organisation. Res. Organ. Behav..

[bib31] Powell A., Rushmer R.K., Davies H. (2009). A Systematic Narrative Review of Quality Improvement Models in Healthcare.

[bib32] Pronovost P., Needham D., Berenholtz S., Sinopoli D., Chu H., Cosgrove S., Goeschel C. (2006). An intervention to decrease catheter-related bloodstream infections in the ICU. N. Engl. J. Med..

[bib33] Pronovost P.J., Demski R., Callender T., Winner L., Miller M.R., Austin J.M., Berenholtz S.M. (2013). Demonstrating high reliability accountability measures at the Johns Hopkins Hospital. Jt. Comm. J. Qual. Improv..

[bib34] Russell G.K., Jimenez S., Martin L., Peake M.D., Woolhouse I. (2014). A multicentre randomised controlled trial of reciprocal lung cancer peer review and supported quality improvement: results from the improving lung cancer outcomes project. Br. J. Cancer.

[bib35] Schensul S., Schensul J.J., LeCompte M.D. (1999). Essential Ethnographic Methods: Observations, Interviews and Questionnaires.

[bib36] Smith H.A., McKeen J.D., Holsapple C.W. (2004). Creating and facilitating communities of practice. Handbook on Knowledge Management.

[bib37] Vangen S., Huxham C. (2003). Enacting leadership for collaborative advantage: dilemmas of ideology and pragmatism in the activities of partnership managers. Br. J. Manag..

[bib38] Wenger E., Snyder W.M. (2000). Communities of practice: the organizational frontier. Harv. Bus. Rev..

[bib39] Yin R. (2003). Applications of Case Study Research.

